# Opinion: Which animals have personality?

**DOI:** 10.1017/pen.2023.9

**Published:** 2024-02-05

**Authors:** Ralph Adolphs, Yue Xu

**Affiliations:** 1 Division of Humanities and Social Sciences, California Institute of Technology, Pasadena, CA, USA; 2 Division of Biology and Biological Engineering, California Institute of Technology, Pasadena, CA, USA

**Keywords:** animal personality, behavior, nonhuman personality

## Abstract

Human personality generally refers to coherent individuating patterns in affect, behavior, and cognition. We can only observe and measure behavior, from which we then infer personality and other psychological processes (affect, cognition, etc.). We emphasize that the study of personality always explains or summarizes patterns not only in behavior but also in these other psychological processes inferred from behavior. We thus argue that personality should be attributed only to nonhuman animals with behaviors from which we can infer a sufficiently rich set of psychological processes. The mere inference of a biological trait that explains behavioral variability, on our view, is not sufficient to count as a personality construct and should be given a different term. Methodologically, inferring personality in nonhuman animals entails challenges in characterizing ecologically valid behaviors, doing so across rich and varied environments, and collecting enough data. We suggest that studies should gradually accumulate such corpora of data on a species through well-curated shared databases. A mixture of approaches should include both top-down fit with extant human personality theories (such as the Big Five) as well as bottom-up discovery of species-specific personality dimensions. Adopting the above framework will help us to build a comparative psychology and will provide the most informative models also for understanding human personality, its evolution, and its disorders.

## 1. What is animal personality?

Personality is a psychological construct commonly used to explain relatively temporally stable individual differences in how a person thinks, feels, and behaves (Roberts & Mroczek, 2008; Coon & Mitterer, 2012; Baumert et al., 2017). Nonhuman animals also exhibit stable individual differences in behavior, but it remains a challenge how to infer their thoughts and feelings. These conceptual differences are reflected in differences in method: for people, we can assess personality with self-report questionnaires (which may of course turn out to be inaccurate or invalid), as well as from behavioral observation; for nonhuman animals, we have only the behavioral observation. Like all psychological processes, we think of personality as a latent variable that cannot be observed directly, but that must ultimately be inferred from behavior. It is important to stress that both lay and scientific conceptions of human personality generally encompass individual differences in a range of psychological processes: “personality is an abstraction used to explain consistency and coherency in an individual’s pattern of affects, cognitions, desires and behaviors” (Revelle, 2007, p. 37).

By contrast, definitions of personality as applied to animals generally restrict themselves to patterns of behavior: personality refers to “between-individual differences in behavior that persist through time” (Carter et al., 2013, p. 467), or “labile behavioral traits that tend to differ consistently between individuals of the same species” (Maskrey et al., 2021, p. 13). We argue here that personality needs to be inferred when we want to explain behavioral patterns so complex that we need to infer psychological terms: these behavioral patterns cannot be explained efficiently by biology alone. For example, when we use “extraversion” in personality to explain or predict a person’s behavior, it will be much more efficient than describing the biological/neurological mechanisms of the behavior (of course, these biological/neurological mechanisms can also be explained by chemistry and physics, which would be the least efficient). To be sure, we would see all psychological processes ultimately as biological (and, for that matter, chemical and physical), but these disciplines have different terms, different explanatory aims, and offer different efficiency in their explanation. According to our view, we do not need to attribute personality to a sea anemone, disagreeing with Briffa and Greenaway (2011), Hensley et al. (2012), and Maskrey et al. (2021). Of course, there are phenotypic traits that show stable individual differences, such as eye color, fur color, or the general appearance of an animal or person. Similarly, there are behavioral traits that show stable individual differences, such as stable differences only in running speed, reaction time, a limp on one side, or a tremor. These examples of traits have biological and neurological explanations, respectively. They do not need psychological explanations. Personality is, in our view, first and foremost, a psychological term and it is always concerned not only with behavior but also with other psychological processes; different terminology should be applied to explanations that are purely biological or neurological.

Indeed, personality should not even be applied to cases where there are individual differences in only one or a narrow set of psychological processes; it needs to be more comprehensive than that. For instance, individual differences in memory or attention alone are just that — like individual differences in running speed, we do not need to attribute personality: we can just say they are individual differences in memory, attention, or running speed and leave it at that. Occam’s razor should be applied here: explanation with a personality trait should be reserved for those cases that would be incomplete otherwise.

No doubt the view described above will be persuasive in attributing personality to only some species (like humans, monkeys, dogs, and probably rodents), exclude it from others (like sea anemones), and leave a lot in a gray zone where debate and further studies are needed (like fish or octopuses). Even sea anemones might turn out to have personalities (Briffa & Greenaway, 2011; Hensley et al., 2012; Maskrey et al., 2021) – but, on our view, we would need a lot more evidence than what is currently offered.

To summarize this section: personality can be inferred from relatively temporally stable patterns of complex behaviors that distinguish individuals from one another. However, we stress that the behavior should be complicated enough that personality is used to explain patterns not only in behavior but also in at least some other psychological processes (like emotion or attention or cognitive control – which, again, we here view as latent variables inferred from behavior). Of course, experiments could be devised to use relatively simple behavioral readouts of psychological processes including personality (e.g., pushing buttons to answer self-report questions). But the key point is that personality should fit into an animal or a person’s psychology that relates personality, affect, and cognition to one another and to behavior – in essence, personality should be an ingredient in a theory about a person’s or animal’s mind that is used to explain behavior more comprehensively. Our view differs from that of some others on animal personality, who have focused only on behavior and not prominently included a need for other psychological processes (Kralj-Fišer & Schuett, 2014; Kaiser & Müller, 2021). How much of a psychology would be required is an open question – but we would urge that there should be several other psychological processes we infer in an animal and that there are relations between these and personality. If it is only behavior that is to be explained, we would suggest using a different term than personality.

## 2. Criteria for animal personality

What observational criteria could be used to infer personality in nonhuman animal species? As Table 1 already shows, quite a variety of approaches have been used, generally requiring substantial interpretation by human observers. The settings vary from the laboratory to zoos to naturalistic environments and immediately raise two critical questions. First, how should behaviors be classified or coded? Second, how much observational data do we need to do this? These two challenges are crucial to identify ethologically meaningful and generalizable patterns in behaviors whose regularity across time is the basis for inferring personality traits. These challenges have been discussed at length by Uher (2008). Building on this discussion, we would emphasize three critical ingredients: collect as complete a dataset over as extended a time as possible; code the data by expert consensus in light of the best knowledge that we have of the animal’s ethology; and collect the data in rich, naturalistic settings. The last point raises a further issue: context dependency.

**Table 1.** A nonexhaustive list of research on animal personality after 2001. See Gosling (2001) for a list of research on animal personality before 2001. See Kralj-Fiser and Schuett (2014) for a list of additional invertebrate studies

Focus of the study	Species	Sample size	Personality traits	Study/References	Studying environment	Animal behaviors measured
Relation between chimpanzee subjective well-being and six established chimpanzee personality factors	Chimpanzee	128	Subjective well-being	(King & Landau, 2003)	Zoo	Zoo workers’ assessment of pleasure derived from social interactions, balance of positive and negative moods, success in goal attainment, and the desirability of being a particular chimpanzee.
Test cross-national generalization of personality structure	Chimpanzee	117	Dominance, extraversion, agreeableness, dependability, emotionality, and openness	(King et al., 2005)	Zoo and sanctuary	Human raters rated chimpanzees using a questionnaire of 43 adjectival personality descriptors.
Relate personality factors to behaviors in different social contexts	Chimpanzee	49	Dominance, extraversion, agreeableness, dependability, emotionality, and openness	(Pederson et al., 2005)	Zoo	Human raters rated chimpanzees using a questionnaire of 43 adjectival personality descriptors. Frequencies of 25 specific behaviors were independently recorded.
Identify robust individual behavioral profiles	Brown and sloth bear	5	The Big Five: openness, conscientiousness, extraversion, agreeableness, neuroticism	(Pastorino et al., 2017)	Zoo	Zookeepers completed questionnaires of 22 adjectives describing bear behaviors.
Relate personality trait to weight gain	Dairy calf	76	Feeding rate and meal frequency	(Carslake et al., 2022)	Farm	Personality trait indicated by feeding behavior (feeding rate and meal frequency – two mathematically orthogonal behaviors)
Analyze effects of age and experience on behavioral development	Dairy calf	28	Boldness, novelty seeking	(Lauber et al., 2006)	Farm and specially built apparatus	Behavior differences due to age: novel object test, startle test, discrimination learning to locate milk feeder
Compare behavior coding based on test batteries and subjective rating based on questionnaire studies of dog personality	Domestic dog	100	Stranger-directed sociability, activity, aggressiveness, trainability	(Mirkó et al., 2013)	In situ and in a designated park	FIDO personality test for dogs (behavior coding), subjective rating given by dog owners
Comparative study of personality judgments on both dogs and their owners, measure accuracy on internal consistency, consensus, and correspondence	Domestic dog	78	Canine analogs of four of the five human five-factor model factors: energy (extraversion), affection (agreeableness), emotional reactivity (neuroticism), and intelligence (openness)	(Gosling et al., 2003)	In situ and in a designated park	Study 1: Subjective ratings given by dog owners and owner’s friend using the Big Five Inventory (BFI) modified for canine behavior. Study 2: behavioral field testing rated by independent observers following behavioral markers of BFI. Study 3: independent judges rated their impressions of each dog based solely on the photographs of the dogs.
Relate leadership to personality differences	Barnacle goose	18	Boldness, dominance, leadership	(Kurvers et al., 2009)	Laboratory and outdoor housing	Open-field test, activity test, novel-object test, leadership test (order of the movement of individuals in pairs towards a feeding patch)
Measure test-retest reliability of behavioral tests	Quail	46	Fearfulness	(Miller et al., 2005, p. 200)	Laboratory	Emergence test, novel object test, novel food test, predator surprise test
Comparison across multiple species	Rat, gerbil, mouse, ferret, dog, cat, domestic fowl		Emotionality, fear, gregariousness, and exploration	Reviewed by Walsh & Cummins (1976)		Open-field test: behaviors of individuals when introduced into an arena, usually novel
Examine the sources of individual variation in antipredator behavior	Iberian rock lizard	34	Boldness	(López et al., 2005)	Laboratory	Antipredator behavior, activity level and refuge use in both low and high risk of simulated predatory attacks in laboratory environment
Relate prior experience to behavior alteration	Rainbow trout	110	Boldness	(Frost et al., 2007)	Laboratory	Latency to approach a novel object, social learning
Identify population-level difference in behavioral syndromes	Three-spined stickleback		Exploration-avoidance, aggression, general activity	(Dingemanse et al., 2007)	Ponds	Exploration-avoidance of novel foods, novel environments, and altered environments
Relate boldness to body size	Poeciliid fish (Brachyrhaphis Episcopi)		Boldness	(Brown & Braithwaite, 2004)	Laboratory	Time to emerge from a shelter and explore a novel environment (positively correlated with body size)
Relate context change to behavioral unpredictability and personality	Beadlet anemone (Actinia equina)	216	Boldness	(Maskrey et al., 2021)	Laboratory	Immersion response (latency to re-extend feeding tentacles with submergence after 30-min emersion) and startle response (latency to re-extend feeding tentacles after a water discharge at the oral disc)
Identify personality from high repeatability of behavior	Beadlet anemone (Actinia equina)	65	Consistent difference in startle response	(Briffa & Greenaway, 2011)	In situ	Startle response
Relate habitat choice to personality	Giant sea anemone (Condylactis gigantea)	135	Boldness	(Hensley et al., 2012)	In situ	Response to disturbance (time for tentacles to relax into their original state after being touched by a model blue crab)

The issue of context dependency offers some valuable criteria for animal personality. Personality constructs need to refer to individual differences that generalize to some extent across time, that is, they should be temporally stable. On the other hand, we would argue that the behaviors from which we can infer the personality constructs need to be context dependent (context sensitive) to some degree (again, contrary to some views in Kaiser & Müller’s (2021) work that have instead stressed contextual consistency). In humans, the context sensitivity of how personality is expressed in behavior has been a well-known feature ever since Walter Mischel’s seminal work (Mischel, 1996).

The inferred personality constructs from the behaviors must have consistency across contexts, otherwise the criterion of temporal stability would be violated as context changes over time. To operationalize this, the consistency merely needs to apply at the level of the inferred personality constructs from recurring patterns of behaviors – not the precise bodily movements themselves. Indeed, behaviors that are too rigid across very different contexts may be inappropriate indicators of personality as we are defining it – they may instead be like sea anemone traits or Parkinsonian tremor in humans: biological traits that are not psychological. Some flexibility and plasticity seem essential (Dall et al., 2004). We would speculate that more open-ended contexts, such as complex social situations, might reveal behavioral differences that can best be explained by personality (Gosling, 2001; Uher, 2008). An important methodological point would thus be to study personality with an emphasis on those situations/contexts where personality actually has a measurable role in explaining and predicting behavior.

The behavioral patterns from which we infer personality in animals, as in humans, should exhibit broad, situation-dependent (context sensitive) variability. This context- or situation dependence is precisely the evidence that personality processes (together with other psychological processes) are interposed between the situational stimuli and the behaviors that they elicit. The other psychological processes, such as affect and cognition, together with environment/context, constitute an important aspect of personality according to many views (Revelle, 2007; Wilt & Revelle, 2015; Arden et al., 2016; Baumert et al., 2017; Renner et al., 2020). For example, a person being an extrovert (as referring to a temporally stable personality trait) will behave differently depending on the situation, and on what mood they are in, whether they are tired or attentive, etc. Such a distribution of the varied behaviors could fruitfully be thought of along the lines of Fleeson’s whole trait model (Fleeson & Jayawickreme, 2015): we can think of a personality trait as the parameters describing the shape of the distribution of a measurable state/behavior that is expressed transiently in different contexts. How personality expresses itself in behavior, through layers of cognitive and affective processing, accounts for the width of the distribution. Density distributions that are very narrow across very different contexts would argue against an effect of personality because the behavior is insufficiently sensitive to context. On the other hand, too broad density distributions that show extreme variability over context would make it difficult to discover a stable pattern. Animal and human personality should show distributions intermediate between these extremes: temporally stable personality traits are inferred from behaviors that are nonetheless sensitive to different contexts. Of course, it is not merely the density distribution per se, but consistent patterns, and interpretable changes in patterns over time, that are the hallmark of context-sensitive personality (Revelle & Condon, 2015; Revelle & Wilt, 2021).

In addition to measuring behavior across a range of different contexts in which personality can manifest, other major practical challenges for the study of animal personality include accessibility, sample size, and ecological validity. In humans, we derive personality traits across a typically large and representative population, but many animal studies will be limited in both sample size and representativeness (cf. Table 1). For instance, two mice might have somewhat different behavioral patterns, but we would need to study many more before concluding that these are indeed behavioral patterns explained by personality, or merely individual differences due primarily to external environmental factors that do not generalize to other mice. In addition, personality in laboratory mice will depend on the strain and differ from wild animals (Broadhurst, 1975; Blanchard et al., 1998; Crabbe et al., 1999; Augustsson & Meyerson, 2004). It will be helpful for the field to have a common set of approaches (perhaps including common tasks and observational methods (Kaiser & Müller, 2021) as well as common algorithms for analysis, such as tests of reliability and coherence), together with shared databases. This will help accumulate knowledge across larger and larger numbers of animals, and across species. As with other such approaches, funding initiatives aimed at consortia to make this possible would be important. Box 1 summarizes some of the challenges that distinguish the study of animal personality.

Box 1. Criteria for animal personality

1. *Distinguishing personality traits from biological or neurological traits*. We urge that the term “personality” be reserved for traits in organisms with behaviors from which we can infer sufficiently rich set of psychological processes. In principle, “personality” is used to explain individual differences not only in behavior but also in other psychological processes (as is the case in humans). Phenotypic traits like eye color or neurological traits like tremor are not examples of personality traits because they are not psychological.
2. *Accounting for context.* Although personalities may themselves be temporally stable, how they express themselves in behavior in different contexts depends on how they act through other psychological processes. A given personality can influence an animal’s emotion, attention, or decision-making. But so can different contexts, generating distributions of states/measurable behaviors from which personality can be inferred (along the lines of Fleeson’s whole trait theory (Fleeson & Jayawickreme, 2015)).
3. *Not all contexts will be equally informative about personality.* More open-ended and unpredictable situations may elicit behaviors from which personality can best be inferred – but they should not be so open-ended as to bury reliable behavioral patterns in noise. Social contexts may be a particularly good class to use here.
4. *Building a cumulative science of animal personality*. Piecemeal studies make comparisons difficult. We urge that the field adopt standards for measuring behaviors, for analysis, and for validation. Ideally, behavioral measures should span a broad range of contexts over a relatively long time. Analyses should include cross-validation and aim for out-of-sample generalizability. We wish to stress the importance of building towards large-scale and integrated projects that can eventually provide uniform datasets and methods, as has been emphasized in reproducibility efforts across psychology (OPEN SCIENCE COLLABORATION, 2015), ecology (Gould et al. 2023), and neuroscience (Niso et al. 2022).
5. *Weighing theory-driven and data-driven personality models*. Biological theories that draw on ecology, evolution, and neuroscience could scaffold personality theories in animals that can be tested further with data-driven approaches.

## 3. Current personality frameworks

One approach to animal personality applies extant (human) personality theories (Table 2) to animals. There are a number of theories on personality dimensions in humans, from Cattell’s 16 (Cattell, 1945; Heather et al., 2008) to the Big Five (McCrae & Costa, 2008) to Eysenck’s three (Eysenck, 1991), as examples. While there are other criteria that might decide in favor of one of these over another (e.g., biological evidence for the reality of a particular dimension), from a statistical point of view they could be compatible with one another: a full trait space would have as many dimensions as there are items or measures, but any one of a number of dimensionality reductions (using factor analysis or principal component analysis, for instance) could compress this to an arbitrary number of fewer dimensions depending on the desired amount of represented variation. In this view, there is no unique personality space, and translation between different spaces is generally straightforward as long as each of them is based on a sufficiently complete set of measures at the outset (Markon et al., 2005; Ludeke et al., 2019; Bainbridge et al., 2022).

**Table 2.** Theories of human personality, brief description, and rating of applicability to animal studies (0 = not applicable, 1 = applicable with substantial modification, 2 = applicable with minor modification)

Broad theory category	Theory	Brief description	Developed by	Rating of applicability to animal studies
Trait theory	Five Factor Model (the Big Five)	Five broad personality factors: openness, conscientiousness, extraversion, agreeableness, and neuroticism, developed by lexical studies on all trait words with the assumption that “traits are so important in daily life that people will have invented names for all the important ones” (McCrae & Costa, 2008, p. 274). NEO personality inventory along with other inventories were developed to measure these personality factors.	Robert McCrae, Paul Costa, and many other scientists (Digman, 1990; McCrae & Costa, 2008)	2
	16 personality traits	Cattell utilized data from objective tests, self-reported questionnaires, and observations from others and developed 16 personality factors and the 16 Personality Factor test through a multivariate mathematical approach.	Raymond Cattell (Cattell, 1945; Heather et al., 2008)	1
	3 trait continuum	Eysenck summarized personality into a 3-trait continuum: personality-extraversion/introversion, neuroticism/stability, and psychoticism/superego, based on both factor analysis and biology origins (heredity).	Hans Eysenck (Eysenck, 1991)	2
Social cognitive theory	Social learning	Each of the three factors, the person, the environment, and the person’s actions, causally influences the others. There are five basic capabilities that determine a person: symbolizing, vicarious, forethought, self-regulatory, and self-reflective capabilities.	Albert Bandura (Bandura, 1978, 2006)	0
	Expectancy-value theory	People behave according to their subjective expectancy of rewards. People of internal locus of control (compared to external locus of control) believe they can control the rewards and punishments they experience with higher probability.	Julian Rotter (Rotter, 1966)	1
	Socio-analytic theory	Assumes that people are motivated by getting along, getting ahead, and finding meaning. Aims to predict individual differences in peoples’ career success. Personality consists of identity and reputation. Social skill translates identity into reputation and is the key to career success. Measures of reputation from observer evaluation are better than self-ratings of personality to assess personality and predict performance.	Robert Hogan (Hogan & Blickle, 2018)	1
	Whole Trait Theory	Whole traits contain two parts: (1) descriptive traits are the density distributions of states, representing both the “within-person variation in personality states within a distribution, and the between-person variation in parameters of distributions” (Fleeson & Jayawickreme, 2015, p. 10); (2) explanatory traits consist of social-cognitive mechanisms that explain descriptive traits.	William Fleeson and Eranda Jayawickreme (Fleeson & Jayawickreme, 2015)	2
Humanistic theory (a person’s understanding of the world is fundamental to who that person is)	Holistic-dynamic theory	Established the hierarchy of needs, which explains the levels of needs from low to high as physiological, safety, belongingness, self-esteem and status, and self-actualization needs. A person must satisfy the lower levels first to then focus on the higher levels.	Abraham Maslow (Feist et al.,^2021^; Geller, 1982)	1
	Person-centered theory	A person’s behavior depends on the drive for self-actualization. Unconditional positive regard (from parents or loved ones) is one necessary ingredient for achieving self-actualization.	Carl Rogers (G. J. Geller, 1982; Boyle et al., 2008)	0
	A theory that is bridging humanistic theory and social-cognitive theory	Personal constructs are the qualities a person uses to understand and evaluate the world. Personal constructs are shaped by a person’s interpretation of their past experiences. Role Construct Repertory Test (Rep test) is developed to assess people’s personal constructs.	George Kelly (Boyle et al., 2008)	0
(no broad theory category)	Reinforcement sensitivity theory	There are three brain systems that all differently respond to rewarding and punishing stimuli. These brain systems are often associated with corresponding emotions (1) fear proneness and avoidance ↔ fight-flight-freeze system (emotion of fear and active avoidance), (2) worry proneness and anxiety ↔ behavioral inhibition system (emotion of anxiety and cautious risk-assessment behavior), (3) optimism, reward orientation, and impulsivity ↔ behavioral approach system (emotion of anticipatory pleasure)	Jeffrey Gray (Feist et al., 2021)	2
(no broad theory category)	Regulative Theory of Temperament	Is derived from the functional properties of the nervous system described by Pavlov: strength of excitation, transmarginal inhibition, and mobility of nervous process. Emphasizes the biological basis of temperament and the regulatory role of temperament on the energetic and temporal aspects of behavior. Temperament traits include briskness, perseverance, sensory sensitivity, emotional reactivity, endurance, and activity.	Jan Strelau (Strelau 1996, Strelau, 2008)	2
(no broad theory category)	Cloninger model of personality	Different responses to punishing, rewarding, and novel stimuli are caused by interaction of three dimensions: (1) novelty seeking (impulsiveness, correlated with low dopamine activity), (2) harm avoidance (anxiousness, correlated with high serotonin activity), and (3) reward dependence (approval seeking, correlated with low norepinephrine activity).	Robert Cloninger (Cloninger, 1986)	2
(no broad theory category)	Psychoanalytic (psychodynamic) theory	Psyche is an internal structure of the mind and is made up of three parts: the id, the ego, and the superego. Emphasizes the importance of unconscious processes underlying behavior. Psychosexual development has five stages: oral, anal, phallic, latency, and genital.	Sigmund Freud, Carl Jung, Neo-Freudians (e.g., Alfred Adler, Karen Horney, Erik Erikson) (Feist et al., 2021)	0

Given the challenges of collecting sufficient observational data from animals, our overarching recommendation for applying extant theories of human personality to behavioral data from animals is to begin small. One might characterize a particular facet/dimension of personality, such as timidness vs. boldness, and then gradually build out from there to a more complete personality once sufficient data are available. In general, we would recommend sticking with one of the most widely accepted theories on personality dimensions, such as the Big-Five, and then to see if perhaps one or more of the dimensions drop out because they have too sparse observations or they seem to be absorbed by another dimension. As can be seen in Table 1, most studies of animal personality offer a relatively small number of dimensions that are often a subset of those in the Big Five, or an amalgamation of some of its factors. Once a study has undertaken such a characterization of a nonhuman species’ personality, however limited, it would then be important to see how well it predicts behaviors to a future situation and how well it generalizes across time and individuals. Using existing human personality theories as the approach could observe animal behavior and score it along the dimensions of the theory as well as design specific situations or tasks intended to assess a particular dimension. Testing across multiple contexts would also be important, as noted above, and emphasizes the need for comprehensive and well-curated data sharing among studies. In general, these approaches have confirmed that at least some dimensions commonly used to describe human personality also apply to nonhuman animals; for example, studies of chimpanzees and bears (Table 1) have used the Big Five (Table 2).

Another approach comes from the study of animal behavior itself, which also offers theories to explain the behavior, but from the perspective of ethology rather than derivation from theories of human personality. Some examples come from careful studies of animal behavior by trained human observers. When studying boldness and exploration, Fox et al. (2009) discovered that mountain chickadees’ behaviors in their exploration of a novel environment and a novel object are uncorrelated, suggesting two independent personality dimensions and contradicting other studies (Carter et al., 2013). In personality studies of chimpanzees, social dominance and dominance-related activities play a pervasive role, conceptualized as a sixth factor distinct from the Big Five in humans (King & Figueredo, 1997). As can be seen from just these brief examples, the ethological factors typically used to characterize variability in some aspects of behavior tend to differ between species and can differ from those popular in humans.

Extant theories of both human and animal personality as described above all have dimensions that are readily interpretable. But all these approaches raise a nagging worry: they are ultimately very much the design and interpretation of humans. An advantage is that we can understand the dimensions used to characterize animal personality. The worry is that we are anthropomorphizing. This brings us to data-driven approaches that can aim to minimize the risk of anthropomorphizing and that could suggest entirely new personality dimensions we might never have thought of intuitively.

## 4. Data-driven approaches to animal personality

The behavioral data collected for a data-driven approach should be as complete as possible over a long time in varied environments to ensure that all variation in behavior is sampled (Uher, 2008; Uher et al., 2008). By applying a data-driven algorithm on such a large high-dimensional dataset, one can extract factors/dimensions or categories. If based on sufficiently diverse and ecologically valid behaviors, this approach can generate personality dimensions from nonhuman animal data that may be quite distinct from those featured in human personality theories. In principle, this could allow us to obtain species-specific personality dimensions suited to a particular nonhuman species’ behaviors, avoiding anthropomorphism (Uher, 2008).

**Figure 1.**
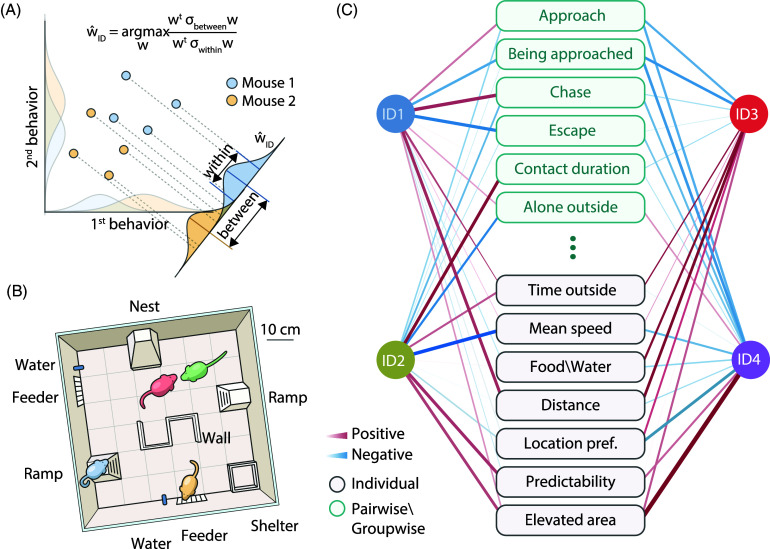
Data-driven inference of personality in mice. **A**. Linear discriminant analysis (LDA) was used for dimensionality reduction by maximizing the ratio of between-subject to within-subject variability. **B.** Schematic of the enriched group-housing environment. **C.** Running LDA on 60 behavioral-feature dimensions (showing 13 representative dimensions) collected from video recordings resulted in four validated identity domains (ID1 - ID4), corresponding to personality dimensions. The IDs themselves are uncorrelated. The width of blue and red connecting lines indicates the strength of the correlation between the four IDs and the 60 behavioral-feature dimensions. For instance, the first personality factor, ID1, is positively correlated with “Chase” and negatively correlated with “Escape”. Adapted with permission from “Identity domains capture individual differences from across the behavioral repertoire” by Forkosh et al., 2009. Copyright 2019 by The Author(s), under exclusive license to Springer Nature America, Inc.

A recent example of such a data-driven approach comes from a study by Forkosh et al. (2019) in mice (Figure 1). Mouse behaviors were recorded over multiple days in a rich environment (Figure 1B). From video recordings, 60 behavioral features were collected based on location tracking, including social behaviors (Figure 1C). Linear discriminant analysis (LDA) (Figure 1A) was applied to reduce the 60 behavioral-feature dimensions into 4 stable so-called “identity domains” (ID, analogous to personality dimensions) by maximizing the ratio of between-mice to within-mouse variability. These 4 identity domains (Figure 1C) were shown to be replicable and stable over developmental stages and social contexts using separate validation data, and the 4 IDs were shown to capture transcriptomic variance in the brain and variance in genetically driven behavioral differences.

Some of the tools developed for data-driven personality studies in animals could fruitfully be applied to humans as well. For instance, densely sampled data are now available from social media, such as smartphone usage. While studies have generally simply attempted to map such data onto extant personality theories such as the Big-Five (Stachl et al., 2020), one could imagine using LDA instead and comparing the results to the four identity domains discovered in the mouse study above.

There may be no clear answer whether to use more “top-down” approaches that start with theorized personality dimensions (as suggested in Section 3 above) or more “bottom-up” approaches that depend on a relatively complete sampling of neural or behavioral data, as suggested in the current section. Uher (2008) has provided a useful taxonomy of the different approaches to animal personality that illustrates the diversity available. Our own preference would be to use a mixture of “top-down” and “bottom-up” approaches, with each being used to inform and revise the other. As we mentioned, a main challenge is the number of samples of observational data available. While semi-automated methods are now making very large datasets from quite diverse situations possible (as in the Forkosh et al., 2019 study) and thus enabling the discovery of strongly data-driven personality dimensions, ecologically based approaches have also been successful in characterizing animal personality. For instance, studies of fear and anxiety have been inventive in constructing situations in the lab that aim to mimic relevant parameters encountered in the wild (e.g., Blanchard & Blanchard, 2008; Kumar et al., 2013; Mobbs & Kim, 2015).

## 5. Concluding comments

The motivations for studying personality in nonhuman animals go well beyond our curiosity and interest in understanding animal behavior *per se*. Animals offer powerful models for investigating the biological and genetic basis of personality (Gosling, 2001), for understanding how it may have evolved (Dall et al., 2004, 2012), and for explaining pathology, topics we have not discussed here for reasons of space. However, we want to stress that neuroscience (and biology more broadly) certainly should figure both in the creation of hypotheses and in the interpretation of results. In this respect, we see biologically inspired theories of personality, such as the line of work from Pavlov through Eysenck to Gray (see Corr & Perkins, 2006, for an overview), as excellent candidates also for animal personality.

Complex behavior in both human and nonhuman animals requires psychology for explanation. We have stressed that animal personality ought to be considered as one component to explain complex behavior. This means that there must be at least some other psychological processes that are also influenced by personality and contribute to explanation of complex behavior, analogous to the “thinking” and “feeling” that figure in theories of human personality. We think that these other psychological processes will include processes such as affect and cognition, but it is important to stress that we simply do not yet have a full theory of animal psychology available. Many more studies will be needed to thoroughly characterize animal behavior in rich environments. Building a mature science of animal personality will go hand in hand with building a mature science of animal psychology.
